# A proof of concept for machine learning-based virtual knapping using neural networks

**DOI:** 10.1038/s41598-021-98755-6

**Published:** 2021-10-07

**Authors:** Jordy Didier Orellana Figueroa, Jonathan Scott Reeves, Shannon P. McPherron, Claudio Tennie

**Affiliations:** 1 Department of Early Prehistory and Quaternary Ecology, University of Tübingen, Tübingen, Germany; 2 Department of Human Evolution, Max Planck Institute for Evolutionary Anthropology, Leipzig, Germany; 3 Technological Primates Research Group, Max Planck Institute for Evolutionary Anthropology, Leipzig, Germany

**Keywords:** Anthropology, Archaeology, Cultural evolution, Computer science, Software

## Abstract

Prehistoric stone tools are an important source of evidence for the study of human behavioural and cognitive evolution. Archaeologists use insights from the experimental replication of lithics to understand phenomena such as the behaviours and cognitive capacities required to manufacture them. However, such experiments can require large amounts of time and raw materials, and achieving sufficient control of key variables can be difficult. A computer program able to accurately simulate stone tool production would make lithic experimentation faster, more accessible, reproducible, less biased, and may lead to reliable insights into the factors that structure the archaeological record. We present here a proof of concept for a machine learning-based virtual knapping framework capable of quickly and accurately predicting flake removals from 3D cores using a conditional adversarial neural network (CGAN). We programmatically generated a testing dataset of standardised 3D cores with flakes knapped from them. After training, the CGAN accurately predicted the length, volume, width, and shape of these flake removals using the intact core surface information alone. This demonstrates the feasibility of machine learning for investigating lithic production virtually. With a larger training sample and validation against archaeological data, virtual knapping could enable fast, cheap, and highly-reproducible virtual lithic experimentation.

## Introduction

Knapped stone tools provide an abundant and long-lasting record of past behaviours and cognition of prehistoric humans on an evolutionary time scale. As a result, the stone artefact record is one of main pillars upon which our understanding of human evolution—and the evolution of human behaviour and cognition—is built. This understanding comes from building inferential links between formal and technological variation observed in the archaeological record and the behavioural, cognitive, and evolutionary processes that lead to its formation^1–8^. However, these links are not always apparent from the stone tools themselves, even in the earliest lithic technologies^8–20^, where the archaeological record is primarily comprised of simpler core and flake tools^10,21–24^. Therefore, archaeologists rely on experimental approaches to replicate stone artefacts under test conditions to determine whether factors such as function^25,26^, raw material availability^27^, skill^28^, technique^29–31^, cognition^28,32–36^, or culture and social learning^33,34,36–38^ played a role in the production (and subsequent discard) of knapped stone tools.

Replication experiments produce insights into the archaeological record but come with some limitations. For one, replication experiments are necessarily affected by the knapper’s own conscious and unconscious biases, their knapping experience, their expertise in the manufacture of certain tool forms, and their range of knowledge of various knapping techniques^39^. In addition, replication experiments cannot be easily reproduced, as many variables cannot be controlled under traditional experimental setups with modern knappers, whilst using a different knapper could introduce an additional variable not under control. Some experimenters partly address these issues by standardising the blanks (i.e. cores or flakes). Standardising raw materials can be done by sawing blocks of material into particular shapes, casting standardised shapes in materials like ceramic or glass, and more recently, by 3D milling of materials into particular shapes. In addition, some experimenters have also begun using machine-controlled knapping, focusing on searching for first principles in knapping by isolating the effect of specific variables on flake production^40–43^. However, standardising blanks and building machine-controlled flaking apparatuses comes with a substantial increase in the amount of time and resources required to prepare, measure, and store the experimental equipment and materials. The need for time and resources is further amplified in the first principles approach, as the number of different experiments needed to investigate the effect of multiple interacting variables is substantial.

One alternative that may circumvent the potentially vast resource and time limitations of traditional lithic experimentation entirely, or otherwise reduce costs, is to develop simulations of stone tool reduction in a digital environment. More specifically, a piece of software able to accurately virtually simulate flaking in three dimensions—comparably similar to actual knapping—would allow for fast, inexpensive, and replicable experiments. Doing so would provide a means to carry out stone tool production experiments in a controlled and reproducible environment for less time and money. The virtual knapping program would also be unaffected by any biases that individual human knappers may have in traditional experiments, and which are hard to control (given also that these biases may in some cases still be unknown).

If knapping could be done virtually, and it were—at least in some cases—a valid substitute for actual knapping, it would serve as a less resource-expensive and more feasible alternative for lithic experiments. Variation in flake shape arises out of a large constellation of parameters that are difficult to systematically test. Having a computer-based model where individual variables could be isolated and examined programmatically would not only increase the speed of what is currently a lengthy process, but could also help us further understand cause and effect relationships of different variables and the interactions between them.

In addition, there would be fewer material requirements, also in terms of long-term storage and transport, since cores could be shaped entirely within a computer, and infinitely duplicated and knapped (and re-knapped), allowing for increased dataset sizes and greater reproducibility. The software could be used to create virtual assemblages testable against actual lithic experiments, examining the influence of certain variables during lithic reduction, or more exhaustively uncovering the possible range of variability of specific reduction techniques. Moreover, the reproducibility and robusticity inherent within a well-made virtual knapping program could even counterbalance some of the error during simulation. A well-crafted virtual knapping program would also be free of human knapper biases entirely, allowing experiments undertaken with it to be more controlled, more reproducible, and perhaps more representative compared to traditional lithic experiments.

A single virtual simulation would ideally take considerably less time to reduce a set of cores than a human knapper would, and even many measurements on the resulting lithics could be automated and performed at a fraction of the time within the software, given that the products would already be digitised. It would also be much more reproducible than current knapping experiments, especially as the (virtual) knapper’s biases could be kept identical for all experiments. Currently, this is not possible to a similar degree due to factors such as differences across knappers (e.g. different skill levels, different modern traditions of knapping) and even within them (e.g. changing motivation, energy, concentration, learning during the experiment).

Here we provide an attempt for a proof of concept of a framework for a virtual knapper using a machine learning approach based on neural networks applied to programmatically created 3D inputs (cores and flakes). Our approach generated a predicted 3D flake and modified core as an output from an intact (i.e. unknapped) core. Our approach proved capable of reliably and validly predicting the length, width, volume, and overall shape of a flake removal from the surface of a core given the point of percussion. We therefore conclude that we successfully created a proof of concept—pathway—for a virtual knapper.

Predicted flakes from a more complete virtual knapper—e.g. using the approach outlined here—could form the basis for (virtual) lithic assemblages to compare with archaeological data, which could also allow archaeologists to examine how the different knapping variables affect the resulting assemblages, and to examine important inferences on the various biological, environmental, and sociocultural factors that could have played a role in the formation of the archaeological assemblages we find in the present; thus, also informing a large part of our understanding of human evolution.

### Machine learning

Arguably, the most intuitive approach for virtual knapping would be physics-based simulations of conchoidal fracture—a type of fracture underlying stone knapping—that would likely require the use of mathematical methods such as finite element analysis (*FEA*). Although the application of FEA for virtual knapping is an important avenue to explore, simulating conchoidal fractures is a resource intensive process, and even the most recent research uses high-performance cluster computers to run simulations^44,45^, especially if we wished to simulate more realistic—hence complicated—knapping scenarios. Simulations wishing to examine the effects that different reduction sequences have on the resulting assemblages, or whether and how some tool *forms* can come about through the reduction of other *forms*^24,29^ require large amounts of flake removals and changing of knapping variables, making a FEA approach not entirely viable.

However, FEAs are only one of many approaches available to tackle the development of a virtual knapping program. To address all of the requirements we had set forth for a virtual knapper, we chose to base our method on neural networks. In a similar way as to how neural networks have allowed for drastically increasing the resolution of images in a fraction of the time it takes for computers to render them traditionally^46,47^, we sought for our neural network framework to predict a flake removal virtually in a fraction of the time it takes for physics-based simulations.

The primary goal for the virtual knapping program was to be a tool that could reliably perform a virtual replication experiment in a very short time without requiring large amounts of computational resources. To this end, a virtual knapper program should also be able to run on an office computer system, not unlike common agent-based modelling software tools, but it should also accurately simulate real stone flaking—focusing, as a starting goal, on hard-hammer percussion knapping (i.e. flakes removed using a hand-held hammerstone to strike the core) of a single raw material type.

Machine learning is a technique that allows computers to build a model of a set of data automatically by analysing the data and *learning* from it, without requiring the user to manually set-up or adjust the model’s parameters^48,49^. The advantage of machine learning-based modelling is that it allows for the bulk of the computational processing—i.e. the *training* of the machine learning model—to be completed prior to the model’s practical use; normally requiring only a very small fraction of the computing time needed to train the model in the first place.

Machine learning is a broad field, and encompasses a wide range of methods and algorithms. One such family of algorithms are artificial neural networks, which are broadly based on a simplified model of inter-connected biological neurons^50,51^. Artificial neural networks learn iteratively by a process known as *training*: the network makes predictions from the input data, then evaluates the error in prediction with a mathematical function, and adjusts its neurons and the strength of their connections in order to improve future predictions^51^.

Artificial neural networks have gained prominence in recent years, as they are advantageous for highly-dimensional data with large numbers of variables and complex interactions. This advantage is even more important for problems where these interactions are difficult to formulate with traditional statistical modelling, or even when we do not know which variables and interactions are important. For instance, human vision is very good at recognising objects, but programming—or mathematically describing—an algorithm to recognise objects in images would be extremely difficult when done traditionally, but can even surpass human performance in specific scenarios^51,52^. Applications of neural networks include autonomous driving^53^, recommendation algorithms^54^, and computer-aided medical diagnosis^55,56^.

**Figure 1:**
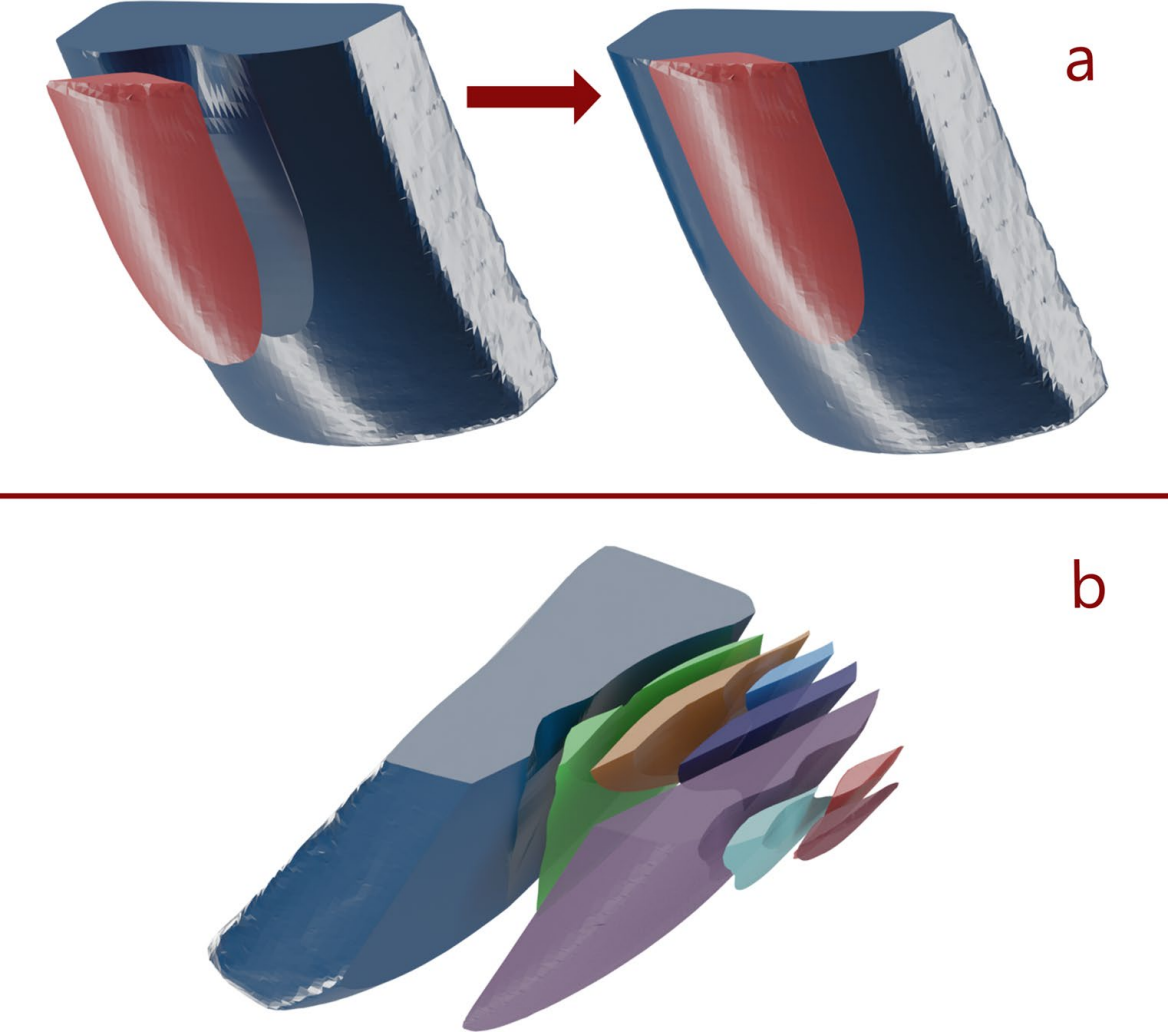
Example of a core and removed flakes from the input dataset. (**a**) The training dataset consists of pairs of flaked cores (blue) and their matching flakes removals (red), oriented such that together they represent the complete core prior to flaking, much like a refit. (**b**) Some of the flake and core pairs were generated in different stages of reduction (see “Methods”). This is an illustration of a generated reduction sequence. Note that, in the dataset, each flake has a matching modified core model as well.

One disadvantage of machine learning, however, is that it often requires a large amount of training data. For our envisioned framework, we required 3D models of a large number of core and flake combinations (i.e. a flake and the core from which it was removed). Such a dataset is not (yet) publicly available, and we did not have the resources to create it ourselves. Moreover, for the initial evaluation of our approach, we sought to avoid adding unnecessary complexity by limiting the shape of the initial cores in our dataset, since—due to the *bias-variance trade-off*—additional variability in a dataset usually requires a larger dataset for the model not to *overfit* to the particular training dataset, performing poorly with new data^51^. In the meantime, we opted instead for programmatically-generated cores and flakes. These have the advantage of being quickly generated with a constrained amount of variability, and if a machine learning model can successfully predict the flakes from this data set, then predicting flakes from a larger more varied data set could likely only be a question of additional training data, as the cores and flakes we used here were based on empirical findings from previous machine-controlled knapping experiments^40^. Unlike previous machine-controlled knapping experiments, however, our flakes were not restricted to a single removal for each core, as we also removed flakes from already knapped cores during data generation (see Fig. 1).

#### Image-to-image translation

Neural network algorithms that predict one 3D shape from another are rare or remain limited in their application^57,58^. However, predictions from 2D datasets are far more common. Here, we circumvent this problem by representing our 3D datasets as a two dimensional surfaces to apply image-to-image translation.

Image-to-image translation is a task in which a neural network model converts (or *translates*) one type of picture to another type altogether. Examples include converting a picture of a landscape taken during the day into a picture of the same landscape at night, converting a line drawing into a photorealistic image, predicting the colourised version of a black and white image, or converting a diagram of a façade into a photorealistic image of a building.

However, since our input consisted of 3D objects, not (2D) images, we needed to encode the information of the relevant surfaces of the 3D cores and flakes into an image. In order to accomplish this task, we made use of depth maps on our 3D cores and flakes.

#### Depth maps

**Figure 2:**
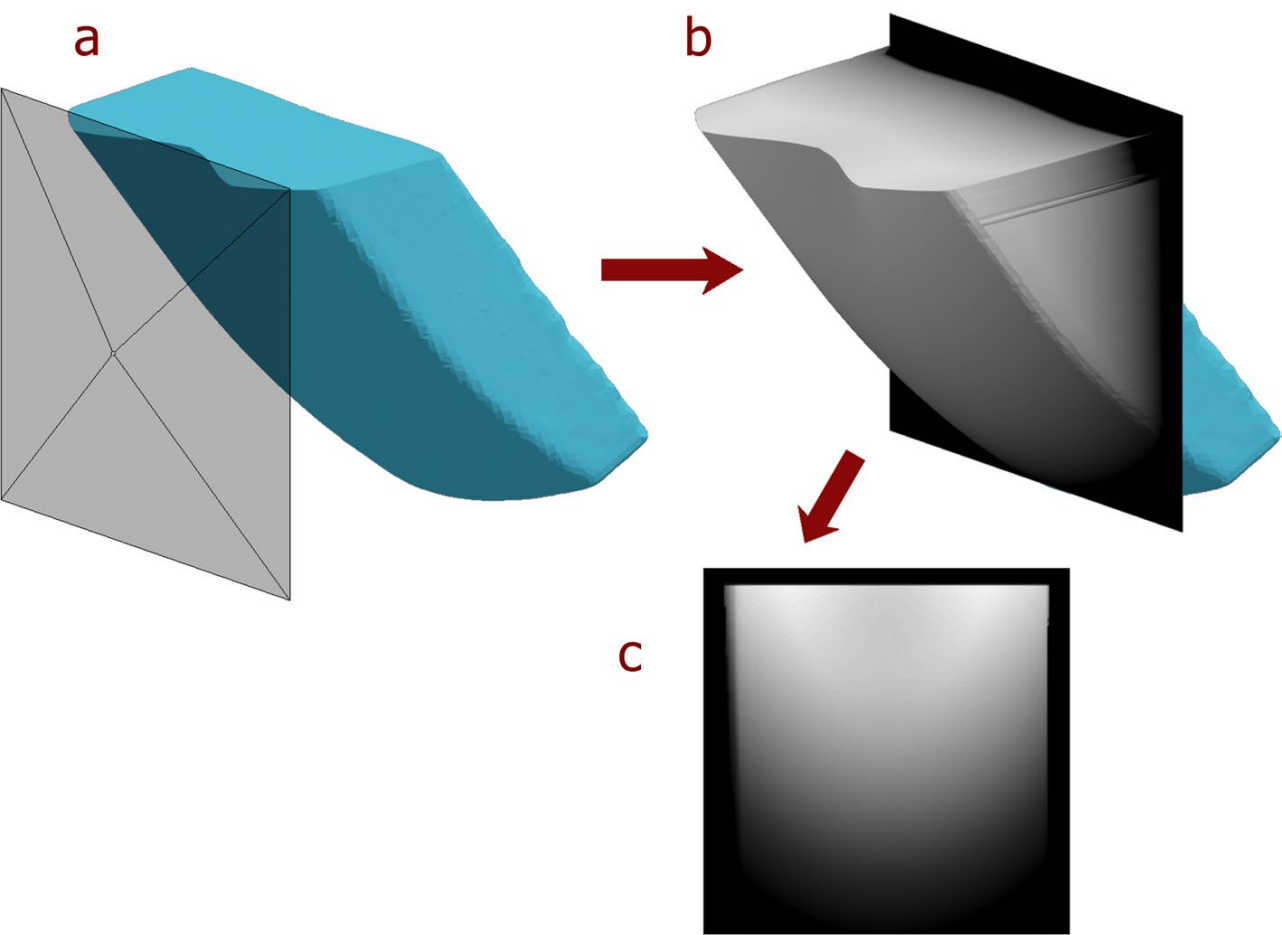
Example of the standard orientation for depth map capture as displayed using a 3-D model of a knapped core, which has a near-perfectly flat platform surface. Note that the platform surface is aligned horizontally with respect to the depth map. Note also that, though difficult to see, the point of percussion is aligned to be in the exact centre of the image. (**a**) 3-D mesh of the core with camera (left) to capture depth map image. (**b**) Depth map rendered into a 3-D surface superimposed over the original core mesh. The depth map’s frame is located at the maximum depth we set when captured. Anything deeper than the maximum depth is rendered as pure black in the image. (**c**) Resulting depth map image.

Depth maps (or *z-buffers*) are images that encode the distance (or *depth*) between a view point in 3D space from where the depth map is captured, and the 3D surfaces visible from that same point (see Fig. 2). Depth maps are very similar in concept to digital elevation models, which capture the elevation of a portion of the Earth’s surface (a 3D property), and encode it into a 2D image whose colours (or raster values) represent different elevations. Depth maps can be conceptualised as a less-restricted form of elevation maps, with the depth map’s maximum allowed depth analogous to the lowest surface elevation of a digital elevation model, and the distance between the surface of the object and the view point as analogous to the elevation of the terrain’s surface.

#### Conditional generative adversarial network (CGAN)

The conditional generative adversarial network (CGAN) architecture consists of a *discriminator* model, which learns to distinguish between the real outputs of our dataset and fake outputs created by a *generator* model, the second part of the CGAN. The generator model learns to create outputs that are realistic enough to fool the discriminator into believing they are real based on the input images. The training process becomes an iterative adversarial contest in which, as the training progresses, the generator becomes better at fooling the discriminator, and the discriminator, in turn, becomes better at detecting the generator’s predicted output. The training ideally culminates in a generator model trained to create outputs that are as close to the real outputs as possible, and able to provide highly accurate predictions under non-training circumstances.

The CGAN performs image-to-image translation by mapping the unmodified core depth maps (input) to the resulting flake volume depth maps (output); what is, in essence, an abstraction of the task of predicting flakes from cores. The predicted flake depth maps obtained as outputs can be then used to obtain the modified core depth map, and with these, calculate the 3D flakes and modified cores using the 3D model of the unmodified core (which would be available in a standard use-case).

## Results

The CGAN predicted the depth maps of the flake volumes removed (n = 603) in under 2 minutes, giving an average of less than 200 ms per individual flake prediction. The length, width, volume, and flake shape error calculation of all the predicted depth maps took less than 3 s, giving an average of less than 5 ms per individual prediction (see “Methods” for information on workstation specifications). The CGAN obtained a high degree of accuracy in all measured metrics.

**Figure 3:**
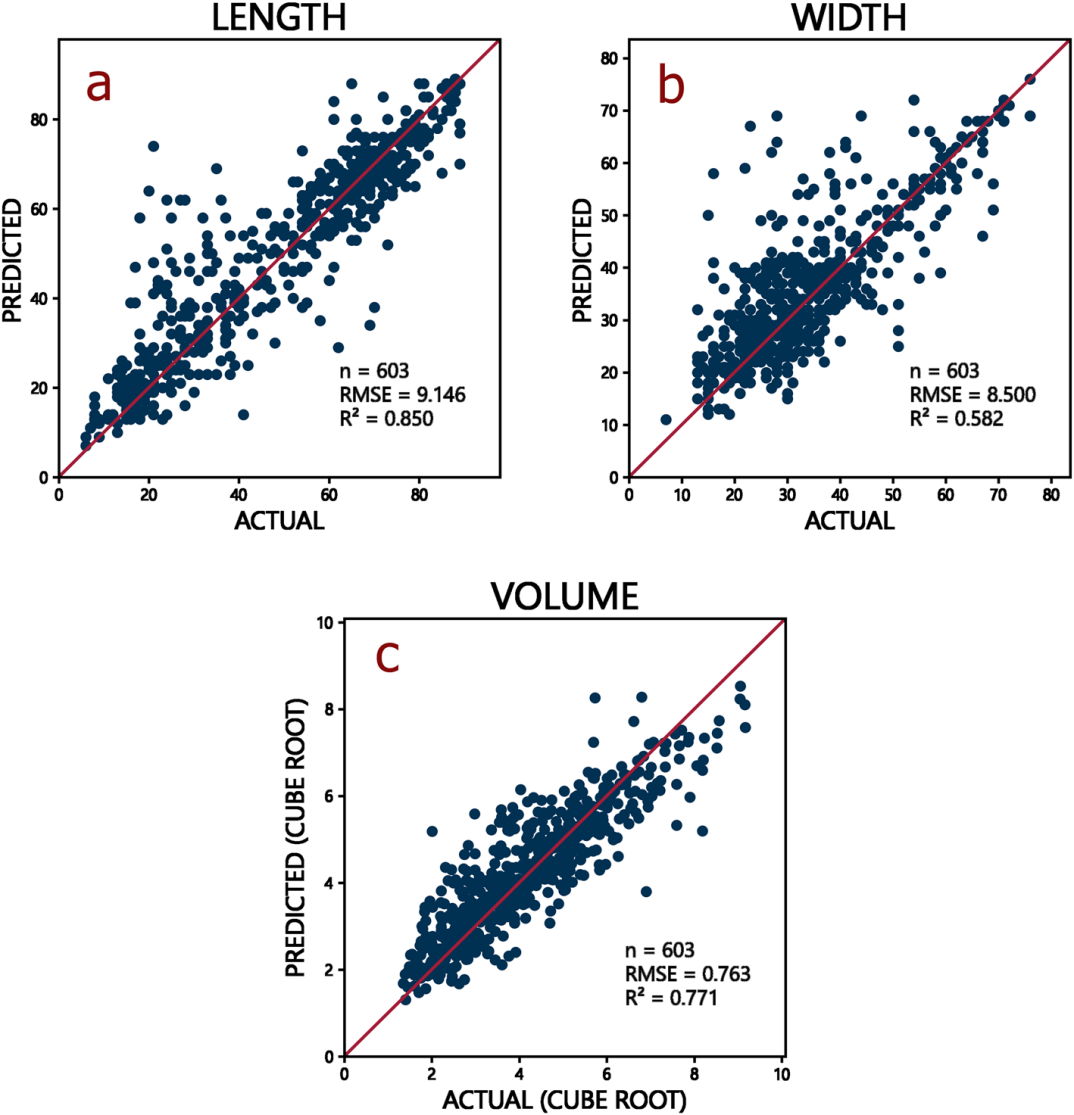
(**a**) Plot of predicted length vs actual length of testing dataset flakes. (**b**) Plot of predicted width vs actual width of testing dataset flakes. (**c**) Plot of predicted cube root of volume vs actual cube root of volume of testing dataset flakes.

An R^2^ of 1.00 (higher is better) and root-mean squared-error (RMSE; see “Methods”) of 0.00 (lower is better) would indicate perfect prediction accuracy. For its prediction of flake length, our model obtained an R^2^ of 0.85, with an RMSE of 9.15 pixels (see Fig. 3a), but a lower R^2^ of 0.58 for its prediction of flake width, with an RMSE of 8.50 pixels (see Fig. 3b). The prediction of the flake’s cube root volume obtained an R^2^ of 0.77 with an RMSE of 0.76 (see Fig. 3c; see “Methods” for the lack of unit of measurement), indicating a high prediction accuracy by the CGAN.

In terms of flake shape prediction, we calculated an average mean absolute error (MAE; see “Methods”) of 0.024 across all flake predictions. The interval for the data (the range of all *possible* values) was [0, 1], which suggests very low error across predictions. Even when considering the interval for the *actual*—rather than the *possible*—data values of our testing dataset ([0.00, 0.75]), or that of our prediction dataset (i.e. [0.00, 0.52]), the average error remained considerably low, at less than 5% of the interval.

**Figure 4:**
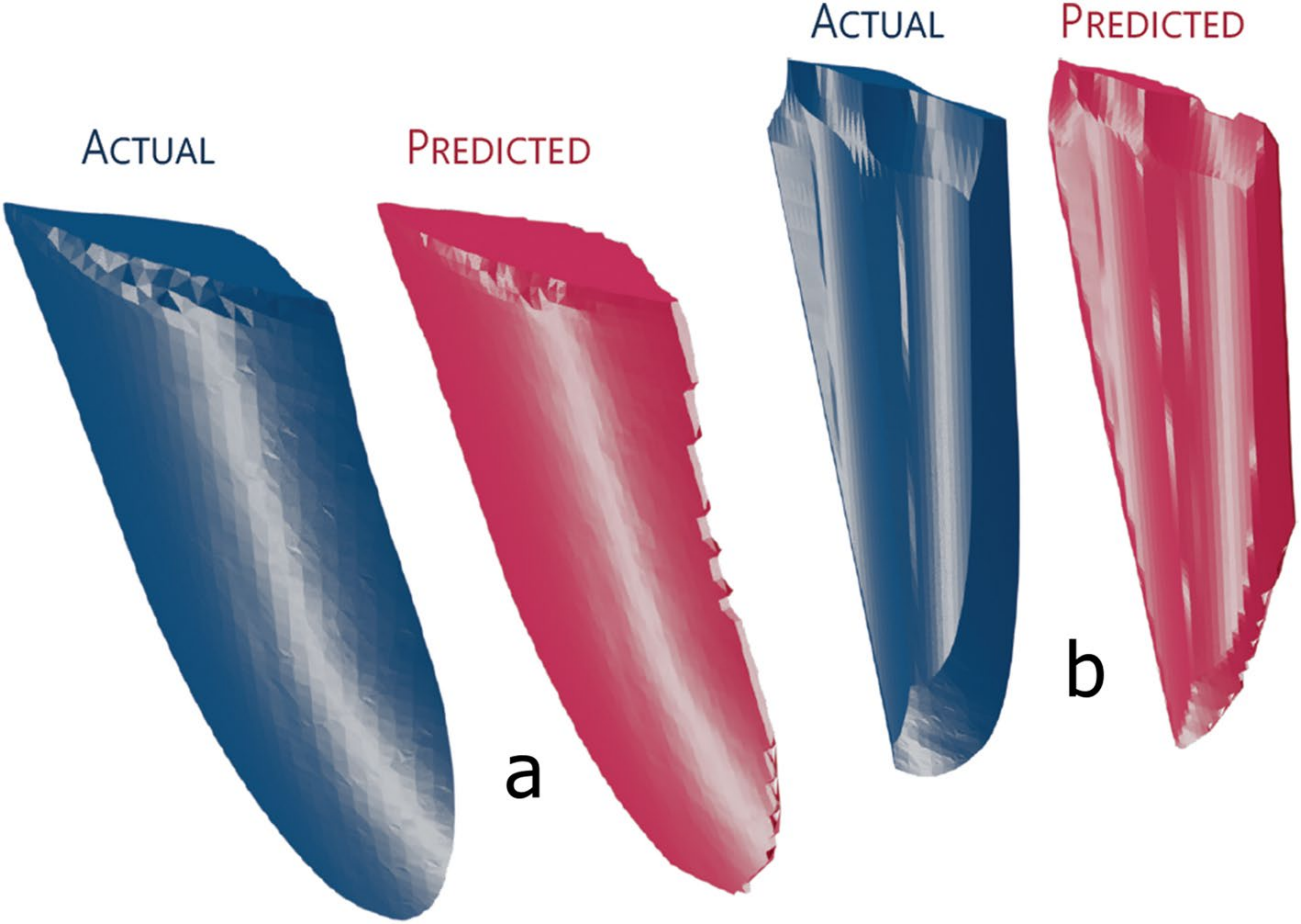
Comparison of two actual vs predicted flakes (**a**,**b**). Note that the size and depth of the predicted flake model was manually scaled to match the size of the actual flake model, though this does not alter the overall shape of the flake (see “Methods” section).

We obtained a very low average RMSE of 0.028 across all flake predictions, but the average normalised root-mean squared-error (NRMSE; ﻿see “Methods”) was higher, at 0.213, or 21.3%. The higher value of the NRMSE is expected due to the way it was calculated, which would weigh errors in smaller flakes proportionally much higher than the same amount of error in more voluminous flakes. Our alternate NRMSE calculation (NRMSE_2_), calculated across all flakes, rather than the average of individual NRMSEs (see “Methods”) had a much lower value of 0.037. Using visual inspection, we can state that the shape of the predicted flakes had a (qualitative) striking resemblance to their respective original input flakes (see Fig. 4). The generation of the 3D models of the predicted flakes from the depth maps took less than 2 minutes; less than 200 ms per individual predicted flake.

A second, independent, training run on the same workstation obtained very similar results (R^2^ of length = 0.85, R^2^ of volume = 0.74, R^2^ of width = 0.55, average MAE = 0.024, RMSE = 0.028, NRMSE = 0.221, NRMSE_2_ = 0.037).

The model remained reasonably accurate with different training dataset sizes, except in width prediction, where prediction accuracy went down significantly; though this seem to have been related to other issues (see “Discussion”). The lowest results were obtained with the training dataset size of 10% of the total dataset (training n = 201, testing n = 1809), with a flake length prediction R^2^ of 0.66, and RMSE of 13.26; a flake width prediction R^2^ of 0.06, with RMSE of 12.98, and cube root of flake volume prediction R^2^ of 0.20 and RMSE of 1.010. We also calculated an average MAE of 0.036, an average RMSE of 0.044, an average NRMSE of 0.314 (or 31.4%), and an NRMSE_2_ of 0.056.

## Discussion

Lithic replication experiments are an important component of human evolutionary research, but replication experiments require considerable material, storage, and time resources to be effective, and being subject to human biases and differences between and within knappers, these experiments become difficult—if not impossible—to reproduce. Even when knapping experiments are replicated, their validity may be affected by knapper’s biases and differences. Here we have used machine learning and programmatically-generated core and flake inputs to produce a proof of concept for a virtual knapping program. Such a program would improve the reproducibility of experimental replication studies by being conducted in a digital environment. In addition, by removing a large portion of the biases (and differences between knappers) brought about by the use of human knappers for replication experiments, a virtual knapping framework could allow researchers to more easily examine the influence of different knapping variables and their interactions in shaping the archaeological lithic assemblages; experiments that would be much more prohibitive to undertake in a real-world environment, even with real-life machine-knapping experiments. Moreover, with a singularly-biased computer model, such experiments would be much more controlled and scientific, as the results would not be biased by human factors (e.g. the knapper’s mood, stamina, motivation, or even different knappers), which could even allow researchers to examine the effect of knapper biases and differences between knappers on lithic reduction.

With the accurate results of our proof of concept framework, we can start evaluating the performance and efficacy of the approach on more complex datasets that better approximate the real world. However, while it is true that the core shapes used varied primarily in the exterior platform angle (the angle between the platform where the flake is struck and the core surface where the flake is removed), some flakes were taken from an initially smooth core surface and some flakes were taken from a core surface made irregular by the removal of previous flakes. Irregular core surfaces are more like those found in the vast majority of actually knapped cores. The next step for the evaluation of the framework is to build a model based on actual core and flake pairs, which will first require a large investment in 3D scanning of material, but will add important variability, and in doing so, will increase the external validity of the model^59^.

This new approach to virtual knapping could also take advantage of what is known as transfer learning, where a model, already trained with a large dataset, can be additionally trained with a similar, albeit more specific and smaller dataset without sacrificing accuracy in prediction. This type of training could be applied to our model, capturing the benefits of the large numbers of realistic data we generated, as well as requiring a lower dataset size for training with actual flakes and cores.

While it is possible that other variables not measured here, or used for the data generation, contribute to the shape of actual flakes, the framework could be extended to incorporate any number of significant new knapping variables either through the acquisition a broader dataset, or through additional neural network models. Striking a core in the same place with the same exterior platform angle but with a different hammer or angle of blow would produce different flakes. If the effects of these other variables were known, then the core and flake data generation program could be made to include them; otherwise, experimental data sets that include these variables would have to be knapped, scanned and included in the model. An additional solution could involve the training of a predictive model specifically for hard hammer percussion and a separate model specifically for soft hammer percussion. Simulation experiments could then be conducted by virtually knapping identical cores with the two separate models to compare their outcomes. Other variables, such as raw material properties, could be tackled in a similar fashion.

We emphasise that this machine learning approach does not intend to fully replace others; rather, it can work in conjunction with other approaches that seek to understand flake formation^40–43,60,61^. The more we can understand flake formation in general, the better we can build a machine learning model to simulate knapping, since we will know which types of variability are important to introduce and which type are not.

Currently, our proof of concept does not yet have the capacity to detect whether a strike would result in a successful flake removal or a failure to detach one. Our data generation assumed successful flaking in all cases; consequently, the model would be over-confident in removing flakes that in actuality would not be possible to remove, adding error during virtual lithic experiments. A simple solution, considering the prediction of the neural network is based on a map of volume removed, is to build a dataset of knapping scenarios where no flake would be detached, and use a blank flake *volume removed* depth map to signal the failure to detach a flake. After training with a dataset that includes failed removals, the model could, theoretically, be able to also predict both failed and successful flake removals.

Based on our results, even with the limitations outlined above, we can conclude that a machine learning-based virtual knapper, using actual knapped 3D cores and flakes as input, is—in principle—a feasible approach to building a complete program for virtual lithic experimentation. This we have showed in our proof of principle study here. The main obstacle to a valid and reliable simulation currently lies in access to high quality core and flake 3D datasets of sufficient size. If a more complete virtual knapping were to prove successful at flake prediction once a sufficiently large and varied dataset of actual cores and flakes was available as input, we would have obtained a framework for widespread, fast, and cost-effective virtual lithic experimentation that could be independently verified as reliable and valid (as this proof of concept was) and become and efficient equivalent to actual knapping. Such a program could also serve as a teaching tool for novice knappers for learning how different knapping variables (e.g. platform depth) affect flake removals. A virtual knapper could be used to perform large-scale lithic experimentation virtually at a fraction of the time and cost, without knapper biases, and would be independently replicable.

## Methods

### Data generation

Using Python 3^62^ and the PyMesh library^63^, we programmatically generated a core and flake dataset. As a starting point, we used a 3D scan of an actual glass core used in controlled machine-knapping experiments^40,42^. We then removed flakes from this core in a manner similar to these controlled experiments. These flakes are simplified versions of the actual flakes removed in^40^, but they conform to the basic properties of flaking and flake morphology. For the initial 405 flake removals, we only knapped one flake from each core, varying platform depths and exterior platform angles. These two variables are known to play a large part in determining flake outcomes^40^, and so by varying them systematically, we were able to produce a variety of flakes.

After the initial 405 flake removals, we also varied the horizontal location along the core edge where the flake was removed. This introduced some asymmetries into the core surface. After an additional 344 flake removals (totalling 749 with the previous 405), we also began removing flakes from already-flaked cores to introduce additional variability in the core surface morphology (see Fig. 1) for an additional 1506 data points.

**Figure 5:**
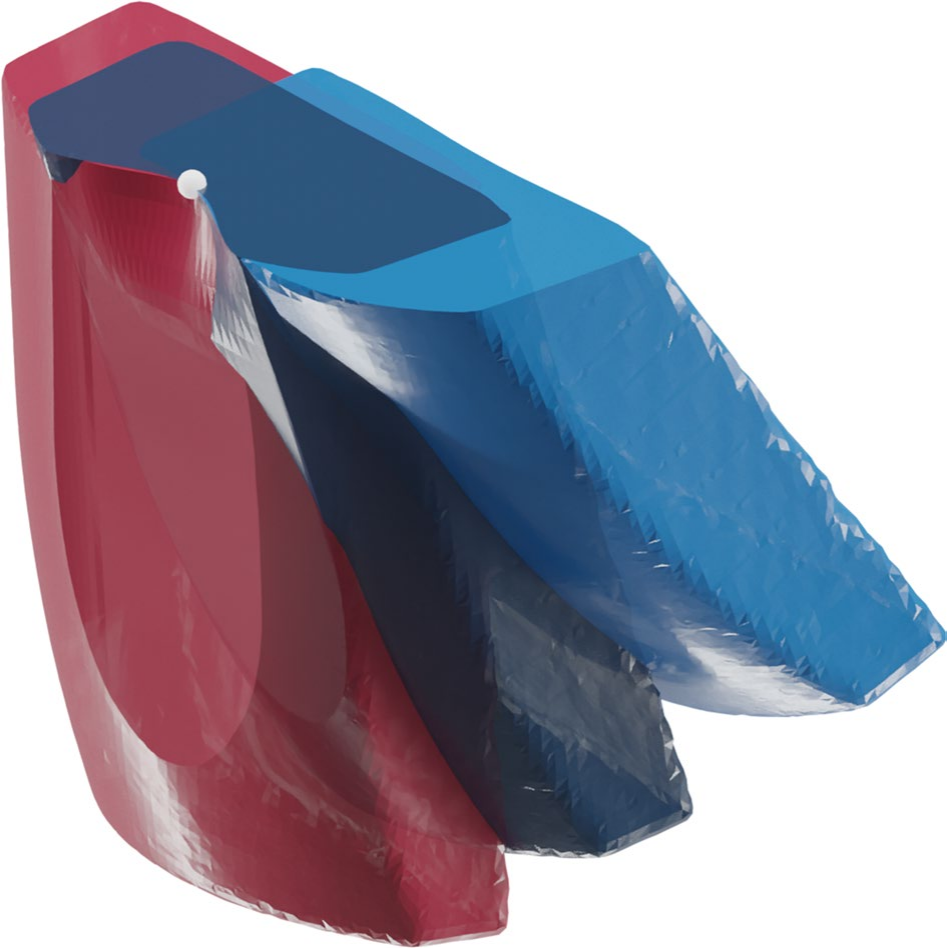
All core and flake models follow a standard orientation, in which their platform surfaces are aligned on the same (horizontal) plane, and all models are centred with the point of percussion (white) in the same location in 3D space. The point of percussion varied by changing the distance from the core edge (platform depth), as well as its horizontal position along the platform edge (off-centre). The differently coloured cores represent cores with different exterior platform angles.

After removing some cases with errors (e.g. missing surfaces, negative platform depth) through a visual inspection and by programming error checks in the depth map generation code (see Supplementary Data SI1), we ended with a total of 2010 sets of 3D models consisting of a modified (i.e. knapped) core and a flake—both positioned and oriented uniformly based on the point of percussion (see Fig. 5), and together forming the unmodified (i.e. un-knapped) core (see Supplementary Data SI5; Fig. 1). All 3D models were stored as .ply files, and the platform parameters for each flake removal were stored as a .csv file.

### Depth map generation

Using Python 3^62^ as well as the Open3D^64^ and NumPy libraries^65^, we captured depth maps of the topology of the 3D core surface, which we could input into a neural network trained for image-to-image translation (see Supplementary Data SI1)—with the assumption that our captured depth maps encoded enough information of the core surface morphology to allow for accurate predictions of resulting flakes by the model. The depth maps were captured with a dimension of 128 × 128 pixels. The depth maps captured the surface from which the flake was detached (i.e. the surface with the flake scar), aligning the platform surface of each core (and flake) perpendicularly to the view point, as well as aligning the point of percussion to be in the horizontal centre—and in the same vertical position—in every depth map. The 3D shapes were projected orthographically to the depth map to avoid angle foreshortening from a perspective projection, in case this was to be detrimental to the model’s prediction accuracy.

In addition, the maximum depth was calculated based on the platform depth and exterior platform angle (all obtained thanks to knowing the location of the point of percussion) to also encode those variables into the depth map itself; the deeper the platform and the more acute the angle, the larger the maximum depth. The depth maps were normalised to an interval of [0, 1], with the maximum depth set to 0, and the point closest to the view point set to a value of 1.

Although the input data only contained already-knapped cores and the last flake removed, the two together were used to generate the depth map of the core prior to flake removal. Since both the flakes and cores were already aligned in 3D space, the core before flaking could be reconstructed.

With the initial core (unmodified) depth map obtained, we calculated a map of the difference between the modified (flaked) and the unmodified core surface, which shows the volume taken from the core by the knapping of the flake. In our model, we used the volume removed as the desired predicted output of our neural network, rather than a depth map of the flake’s ventral or dorsal surface, since the dorsal flake surface is already encoded in the unmodified core depth map, and the ventral surface, in that of the modified core. Thus, we can obtain the shape of the flake removal by calculating the difference between the modified and unmodified core surface depth maps, and we can, in turn, calculate the modified core surface depth map by subtracting the volume removed from the unmodified core surface depth map. In a standard use case scenario, we would only have the unmodified core surface depth map as an input to the neural network model, which would output a predicted volume removed depth map, with which we could obtain the modified (flaked) core surface and the flake removed.

### Neural network training and testing

With the depth maps of our generated cores and flakes, we built a conditional generative adversarial network (CGAN) for image-to-image translation^66^ following the implementation in the TensorFlow documentation^67^ using Python 3^62^ and the TensorFlow 2 library (see Supplementary Data SI2)^68^.

We shuffled the order of our depth map pairs and split our depth map dataset (n = 2010) into two smaller subsets: 70% for the training dataset (n = 1407), and 30% for the testing dataset (n = 603). The training data was shuffled once more when creating the Tensorflow Dataset object.

We trained the CGAN for 150 epochs (see “Supplementary Information S1” for code). Our input was the unmodified core depth maps of the training dataset, and we provided the CGAN with the volume removed depth map as the desired output to learn to predict. The training was done on an Asus Vivobook Pro 17 laptop (N705UD), with a 4-core 8-thread Intel Core i7-8550U CPU, 16 GB of DDR4 RAM, and a dedicated NVIDIA GeForce GTX 1070 GPU. The training process took approximately 2.5 hours using the NVIDIA GPU as a CUDA platform.

After training was completed, we moved to testing the trained model. We input only the unmodified core depth maps from our dataset into our CGAN to obtain a dataset of predicted flake volume depth maps. Prediction for all 603 depth maps took less than 2 minutes total.

#### Data analysis

After converting the 3D models of the cores and flakes into 2D depth maps, splitting these into a training and testing dataset, as well as feeding the latter to our neural network to predict flake removals, we measured the predicted depth maps and compared them with the matching depth maps from our output testing dataset (see Supplementary Data SI3).

To calculate prediction accuracy, we compared the predicted flake volume depth maps with those of our testing dataset. Since our analyses were performed on the depth maps, rather than the 3D objects, the prediction metrics had pixels for units, rather than metric units such as centimetres. We applied common basic quantitative lithic analyses to compare the predicted and testing dataset, and examine the prediction accuracy.

We compared the length, width, and cube root of volume of the flakes across datasets. In order to evaluate the accuracy in predicted flake shape, we calculated the average mean absolute error (MAE), average root-mean squared-error (RMSE), and normalised root-mean squared-error (NRMSE, normalised by the range of values for each testing depth map) between the predicted and actual flake depth map images.

To calculate our metrics, we first set a cut-off threshold to eliminate low-level noise in the predicted depth maps. We used different threshold values (0.1, 0.05, 0.01, and 0.005), but observed that the value of 0.01 provided the best results across all training runs, and was therefore the one used in the reporting of results. We first found all the pixels with values higher than our noise threshold for both testing and predicted flakes, and assigned this area of the image as the flake. For our linear measurements, we used the width and length of this area to calculate flake length and width for both predicted and actual flakes. Therefore, the RMSE for the prediction accuracy for these metrics have pixels as units. To calculate the volume, we summed the *elevation* values of each pixel in the image that was above the noise threshold. It is difficult to assign an actual unit to the depth data, as it is based on abstract and normalised 3D Cartesian distance units; therefore, we reported the RMSE for the volume—as well as the flake shape accuracy metrics—as unit-less.

To prevent artificially reducing the error by using image pixels that contained no data (thus increasing the total number of data points with low values, and reducing the mean error), we calculated the error only for the part of the image that contained either the predicted or actual flake. Areas of the depth map that only had noise or had a value of zero were not used for the calculation. We calculated the difference in each pixel between the predicted and actual depth maps, then calculated the MAE, RMSE, and NRMSE of each flake prediction, with each pixel representing one data point. Once we had obtained the MAEs, RMSEs, and NRMSEs of every individual flake prediction, we calculated the averages for each metric, which we report in our results. Finally, we also calculated a different average NRMSE (NRMSE_2_) by taking the average RMSE previously calculated, and normalising it by dividing it by the range of testing data values (y_max_—y_min_), rather than normalising it per flake prediction.

We additionally calculated the RMSE of the prediction using our own code, as well as the coefficient of determination (R^2^) between the CGAN’s predictions and the testing data using the scikit-learn library’s metrics.r2_score function^69^.

On a reviewer’s request, we performed the calculation of all previously described metrics separately for initial versus subsequent removals (i.e. the first removal from an intact core, and removals from non-intact cores). Since there was no a priori labelling of either initial or non-initial flake removals, JDOF visually inspected all cores and compiled a list of initial flake removals. Although great care was taken to include all initial flake removals—and *only* initial flake removals—there could have been some that were missed, but we considered our labelling was thorough enough that the results would remain valid.

According to the results from these separate analysis (see Supplementary Data SI5), the model had a higher prediction accuracy with initial removals when compared to non-initial removals (e.g. length prediction R^2^ = 0.925 vs. 0.806), even as the initial flakes were less numerous (n = 243) than flakes from subsequent removals (n = 360). The higher accuracy with initial flakes was true for all metrics, save for width prediction, where the prediction for initial removals was considerably lower compared to that of subsequent removals, with an R^2^ of 0.197 vs. 0.596. The pattern was constant for the models trained with different fractions of the data except for the model trained with 10% of the data, which was instead more accurate with non-initial removals (e.g. length prediction R^2^ = 0.785 vs. 0.591). However, for the analysis of the initial flake removals, the width prediction R^2^ was calculation as a negative value (the width prediction for the 10% run was quite low already), which is a possibility with the scikit-learn function used, and suggests that specific model was worse than a constant model.

With the addition of the processing time for the separate analyses, the time taken for the analysis of the 603 predictions was approximately doubled from the original 3 seconds (with the singular analysis) to approximately 6 seconds total (with both the singular and separate analyses).

Finally, using Python 3^62^ and the Open3D^64^ and NumPy^65^ libraries (see Supplementary Data SI4), we transformed the predicted depth maps to predicted 3D models of flakes to perform an additional visual comparison between predicted and actual shape. These analyses were performed in a custom-built desktop computer, with a 6-core 12-thread AMD Ryzen 5 3600 CPU, and 16 GB of DDR4 RAM.

Due to the current depth-mapping algorithm, in order to produce the visualisation in Fig. 4, we had to manually scale down (i.e. make the model smaller in all dimensions) and reduce the depth (make the model smaller in the z-dimension) of the predicted flakes to match the models of their respective actual flake through visual inspection. The resizing process does not affect flake shape, nor its width and length, and serves as a useful visualisation of the possible accuracy of our framework, even if it is not mathematically precise. Future iterations of the program could allow the resizing of the predicted flake 3D model automatically using the precise scale of the 3D model of the actual flake with some modification of the framework’s code. Moreover, the depth map generation could be done using a perspective, instead of an orthographic projection, as we observed that reconstructing the 3D model was more difficult using our remeshing method.

#### Additional tests

We trained and evaluated the CGAN using different training dataset sizes to examine the robusticity of our framework: 10% (n = 201), 30% (n = 603), and 50% (n = 1005); see SI5.

## Data Availability

The dataset generated and analysed during the current study, as well as the code used for the modelling and analysis are available in an Open Science Framework repository: 10.17605/OSF.IO/ANQZF.

## Code availability

The code used for the processing and analysis of the generated dataset are available in an Open Science Framework repository: 10.17605/OSF.IO/ANQZF.

## References

[1] Foley R, Lahr MM (2003). On stony ground: Lithic technology, human evolution, and the emergence of culture. Evol. Anthropol. Issues News Rev..

[2] Wynn T, Hernandez-Aguilar RA, Marchant LF, Mcgrew WC (2011). ‘An ape’s view of the Oldowan’ revisited. Evol. Anthropol. Issues News Rev..

[3] Stout D (2011). Stone toolmaking and the evolution of human culture and cognition. Philos. Trans. R. Soc. B Biol. Sci..

[4] Bar-Yosef O, Van Peer P (2009). The *Chaîne Opératoire* approach in Middle Paleolithic Archaeology. Curr. Anthropol..

[5] Gallotti R, Gallotti R, Mussi M (2018). Before the Acheulean in East Africa: An overview of the Oldowan Lithic Assemblages. The Emergence of the Acheulean in East Africa and Beyond.

[6] Muller A, Clarkson C, Shipton C (2017). Measuring behavioural and cognitive complexity in lithic technology throughout human evolution. J. Anthropol. Archaeol..

[7] Muller A, Clarkson C (2016). Identifying major transitions in the evolution of lithic cutting edge production rates. PLoS ONE.

[8] Dibble HL (2017). Major fallacies surrounding stone artifacts and assemblages. J. Archaeol. Method Theory.

[9] Tennie C, Premo LS, Braun DR, McPherron SP (2017). Early stone tools and cultural transmission: Resetting the null hypothesis. Curr. Anthropol..

[10] de la Torre I (2011). The origins of stone tool technology in Africa: A historical perspective. Philos. Trans. R. Soc. B Biol. Sci..

[11] de Torre I, Mora R, Hovers E, Braun DR (2009). Remarks on the current theoretical and methodological approaches to the study of technological strategies of early humans in Eastern Africa. Interdisciplinary Approaches to the Oldowan.

[12] Braun DR, Hovers E, Hovers E, Braun DR (2009). Introduction: Current issues in Oldowan Research. Interdisciplinary Approaches to the Oldowan.

[13] Barsky D, Hovers E, Braun DR (2009). An overview of some African and Eurasian Oldowan Sites: Evaluation of hominin cognition levels, technological advancement and adaptive skills. Interdisciplinary Approaches to the Oldowan.

[14] Gowlett JAJ (2009). Artefacts of apes, humans, and others: Towards comparative assessment and analysis. J. Hum. Evol..

[15] de la Torre I, Mora R, Domínguez-Rodrigo M, de Luque L, Alcalá L (2003). The Oldowan industry of Peninj and its bearing on the reconstruction of the technological skills of Lower Pleistocene hominids. J. Hum. Evol..

[16] de la Torre I, Mora R (2014). The transition to the Acheulean in East Africa: An assessment of paradigms and evidence from Olduvai Gorge (Tanzania). J. Archaeol. Method Theory.

[17] Corbey R, Jagich A, Vaesen K, Collard M (2016). The Acheulean handaxe: More like a bird’s song than a beatles’ tune? The Acheulean Handaxe: More like a bird’s song than a beatles’ tune?. Evol. Anthropol. Issues News Rev..

[18] McNabb J, Binyon F, Hazelwood L (2004). The large cutting tools from the South African Acheulean and the Question of Social Traditions. Curr. Anthropol..

[19] Shea JJ (2006). Child’s play: Reflections on the invisibility of children in the paleolithic record. Evol. Anthropol. Issues News Rev..

[20] Wynn T, Gowlett J (2018). The handaxe reconsidered. Evol. Anthropol. Issues News Rev..

[21] Kuman K, Smith C (2014). Oldowan industrial complex. Encyclopedia of Global Archaeology.

[22] *Interdisciplinary Approaches to the Oldowan*. (Springer, 2009).

[23] Schick KD, Toth N, Schick KD, Toth NP (2006). An Overview of the Oldowan Industrial Complex: The sites and the nature of their evidence. The Oldowan: Case studies into the earliest Stone Age.

[24] Isaac GL (1976). Stages of cultural elaboration in the Pleistocene: Possible archaeological indicators of the development of language capabilities. Ann. N. Y. Acad. Sci..

[25] Pettigrew DB, Whittaker JC, Garnett J, Hashman P (2015). How Atlatl Darts behave: Beveled points and the relevance of controlled experiments. Am. antiq..

[26] Eren MI (2016). Test, model, and method validation: The role of experimental stone artifact replication in hypothesis-driven archaeology. Ethnoarchaeology.

[27] Braun DR, Tactikos JC, Ferraro JV, Arnow SL, Harris JWK (2008). Oldowan reduction sequences: Methodological considerations. J. Archaeol. Sci..

[28] Khreisheh NN (2013). The Acquisition of Skill in Early Flaked Stone Technologies: An Experimental Study.

[29] Moore MW, Perston Y (2016). Experimental insights into the cognitive significance of early stone tools. PLoS ONE.

[30] Archer W, Braun DR (2010). Variability in bifacial technology at Elandsfontein, Western cape, South Africa: A geometric morphometric approach. J. Archaeol. Sci..

[31] Toth N (1985). The oldowan reassessed: A close look at early stone artifacts. J. Archaeol. Sci..

[32] Putt SSJ, Wijeakumar S, Spencer JP (2019). Prefrontal cortex activation supports the emergence of early stone age toolmaking skill. Neuroimage.

[33] Putt SS, Wijeakumar S, Franciscus RG, Spencer JP (2017). The functional brain networks that underlie Early Stone Age tool manufacture. Nat. Hum. Behav..

[34] Putt SS, Woods AD, Franciscus RG (2014). The role of verbal interaction during experimental bifacial stone tool manufacture. Lithic Technol..

[35] Stout D, Hecht E, Khreisheh N, Bradley B, Chaminade T (2015). Cognitive demands of lower Paleolithic toolmaking. PLoS ONE.

[36] Stout D, Toth N, Schick K, Chaminade T (2008). Neural correlates of Early Stone Age toolmaking: Technology, language and cognition in human evolution. Philos. Trans. R. Soc. B Biol. Sci..

[37] Morgan TJH (2015). Experimental evidence for the co-evolution of hominin tool-making teaching and language. Nat. Commun..

[38] Shipton C, Nielsen M (2015). Before cumulative culture: The evolutionary origins of overimitation and shared intentionality. Hum. Nat..

[39] Pargeter J, Khreisheh N, Shea JJ, Stout D (2020). Knowledge vs. know-how? Dissecting the foundations of stone knapping skill. J. Hum. Evol..

[40] Dibble HL, Režek Z (2009). Introducing a new experimental design for controlled studies of flake formation: Results for exterior platform angle, platform depth, angle of blow, velocity, and force. J. Archaeol. Sci..

[41] Režek Z, Lin S, Iovita R, Dibble HL (2011). The relative effects of core surface morphology on flake shape and other attributes. J. Archaeol. Sci..

[42] Magnani M, Režek Z, Lin SC, Chan A, Dibble HL (2014). Flake variation in relation to the application of force. J. Archaeol. Sci..

[43] Lin S, Režek Z, Braun D, Dibble H (2013). On the utility and economization of unretouched flakes: The effects of exterior platform angle and platform depth. Am. Antiq..

[44] Bilgen C, Kopaničáková A, Krause R, Weinberg K (2018). A phase-field approach to conchoidal fracture. Meccanica.

[45] Kopaničáková A, Krause R (2020). A recursive multilevel trust region method with application to fully monolithic phase-field models of brittle fracture. Comput. Methods Appl. Mech. Eng..

[46] Xiao L (2020). Neural supersampling for real-time rendering. ACM Trans. Graph..

[47] Dong C, Loy CC, He K, Tang X, Fleet D, Pajdla T, Schiele B, Tuytelaars T (2014). Learning a deep convolutional network for image super-resolution. Computer Vision—ECCV 2014.

[48] Hastie T, Tibshirani R, Friedman JH (2009). The Elements of Statistical Learning: Data Mining, Inference, and Prediction.

[49] Fernandes de Mello R, Antonelli Ponti M (2018). Machine Learning: A Practical Approach on the Statistical Learning Theory.

[50] Fukushima K (1980). Neocognitron: A self-organizing neural network model for a mechanism of pattern recognition unaffected by shift in position. Biol. Cybernet..

[51] Aggarwal CC (2018). Neural Networks and Deep Learning: A Textbook.

[52] He, K., Zhang, X., Ren, S. & Sun, J. Delving deep into rectifiers: surpassing human-level performance on ImageNet classification. in *2015 IEEE International Conference on Computer Vision (ICCV)*, 1026–1034 (IEEE, 2015). 10.1109/ICCV.2015.123.

[53] Schwarting W, Alonso-Mora J, Rus D (2018). Planning and decision-making for autonomous vehicles. Annu. Rev. Control Robot. Auton. Syst..

[54] He, X. *et al.* Neural collaborative filtering. in *Proceedings of the 26th International Conference on World Wide Web* 173–182 (International World Wide Web Conferences Steering Committee, 2017). 10.1145/3038912.3052569.

[55] El-Dahshan E-SA, Mohsen HM, Revett K, Salem A-BM (2014). Computer-aided diagnosis of human brain tumor through MRI: A survey and a new algorithm. Expert Syst. Appl..

[56] Ronneberger O, Fischer P, Brox T, Navab N (2015). U-Net: Convolutional Networks for Biomedical Image Segmentation. Medical Image Computing and Computer-Assisted Intervention – MICCAI 2015.

[57] Milletari, F., Navab, N. & Ahmadi, S.-A. V-Net: Fully convolutional neural networks for volumetric medical image segmentation. In *2016 Fourth International Conference on 3D Vision (3DV)*, 565–571 (IEEE, 2016). 10.1109/3DV.2016.79.

[58] Wang, W., Huang, Q., You, S., Yang, C. & Neumann, U. Shape inpainting using 3D generative adversarial network and recurrent convolutional networks. in *2017 IEEE International Conference on Computer Vision (ICCV)*, 2317–2325 (IEEE, 2017). 10.1109/ICCV.2017.252.

[59] Lin SC, Rezek Z, Dibble HL (2018). Experimental design and experimental inference in stone artifact archaeology. J. Archaeol. Method Theory.

[60] Dogandžić T (2020). The results of lithic experiments performed on glass cores are applicable to other raw materials. Archaeol. Anthropol. Sci..

[61] Archer W (2017). A geometric morphometric relationship predicts stone flake shape and size variability. Archaeol. Anthropol. Sci..

[62] Van Rossum G, Drake FL (2009). Python 3 Reference Manual.

[63] Zhou Q (2020). PyMesh/PyMesh.

[64] Zhou, Q.-Y., Park, J. & Koltun, V. Open3D: A modern library for 3D data processing. *arXiv preprint* arXiv:1801.09847 (2018).

[65] Oliphant TE (2006). A guide to NumPy.

[66] Isola, P., Zhu, J.-Y., Zhou, T. & Efros, A. A. Image-to-image translation with conditional adversarial networks. in *2017 IEEE Conference on Computer Vision and Pattern Recognition (CVPR)*, 5967–5976 (2017). 10.1109/CVPR.2017.632.

[67] TensorFlow. Pix2Pix | TensorFlow Core. *TensorFlow Core* https://www.tensorflow.org/tutorials/generative/pix2pix (2020).

[68] Abadi, M. *et al.* TensorFlow: Large-Scale Machine Learning on Heterogeneous Distributed Systems. *arXiv preprint* arXiv:1603.04467 (2016).

[69] Pedregosa F (2011). Scikit-learn: Machine learning in Python. J. Mach. Learn. Res..

